# An IGF2BP2–MYBL2–HMGB2 Signaling Axis Promotes Macrophage Immune Evasion in Hepatocellular Carcinoma

**DOI:** 10.34133/cancomm.0058

**Published:** 2026-09-30

**Authors:** Jin Woong Ha, Sang Yean Kim, Soyoung Jeon, Min Jeong Na, Jung Hwan Yoon, Suk Woo Nam

**Affiliations:** ^1^ Department of Pathology, College of Medicine, The Catholic University of Korea, Seoul, Republic of Korea.; ^2^ Functional RNomics Research Center, The Catholic University of Korea, Seoul, Republic of Korea.; ^3^ Department of Biomedicine & Health Sciences, Graduate School, The Catholic University of Korea, Seoul, Republic of Korea.; ^4^ NEORNAT Inc., Seoul, Republic of Korea.; ^5^ Institute of Hansen’s Disease, College of Medicine, The Catholic University of Korea, Seoul, Republic of Korea.

Macrophages are innate immune effectors that eliminate abnormal cells through phagocytosis, a process regulated by “macrophage checkpoints” that balance pro- and anti-phagocytic signals [1,2]. However, tumor cells exploit these checkpoint pathways to evade clearance and persist within the tumor microenvironment [3]. The cluster of differentiation 47 (CD47)–signal regulatory protein α (SIRPα) axis represents the prototypical macrophage checkpoint, and increasing evidence indicates that cancers exploit such anti-phagocytic mechanisms to promote tumor progression and immune evasion [4]. Although macrophages constitute a predominant immune cell population in hepatocellular carcinoma (HCC), the tumor-intrinsic pathways that regulate macrophage-mediated phagocytosis remain poorly defined.

Herein, we investigated the differential expression of N6-methyladenosine (m6A)-regulatory genes using 4 independent transcriptomic datasets: Catholic_mLIHC, TCGA_LIHC, ICGC_LIRI, and GSE77314 (Table S1 and Supplementary Materials). Insulin-like growth factor 2 mRNA-binding protein (IGF2BP) family members were markedly overexpressed in tumors compared with normal tissues (Fig. S1A), and higher mRNA expression of *IGF2BP1*, *IGF2BP2*, and *IGF2BP3* was significantly associated with more advanced HCC stage (Fig. 1A and Fig. S1B). To identify common RNA targets of the IGF2BP family, we analyzed the RNA immunoprecipitation sequencing (RIP-seq) dataset. Venn diagram analysis revealed 4,137 genes commonly enriched across the IGF2BP family (Fig. S1C). Among the IGF2BP family, only *IGF2BP2* mRNA was significantly associated with poor prognosis based on Kaplan–Meier survival analysis (Fig. S1D). Of the commonly enriched candidates, MYB proto-oncogene like 2 (*MYBL2*) was identified as the most significantly overexpressed downstream target of IGF2BP2 (Fig. S1E and Table S2). *MYBL2* mRNA was markedly overexpressed in HCC and associated with advanced disease stage and worse survival (Fig. 1B and C and Fig. S1F to K).

**Fig. 1.**
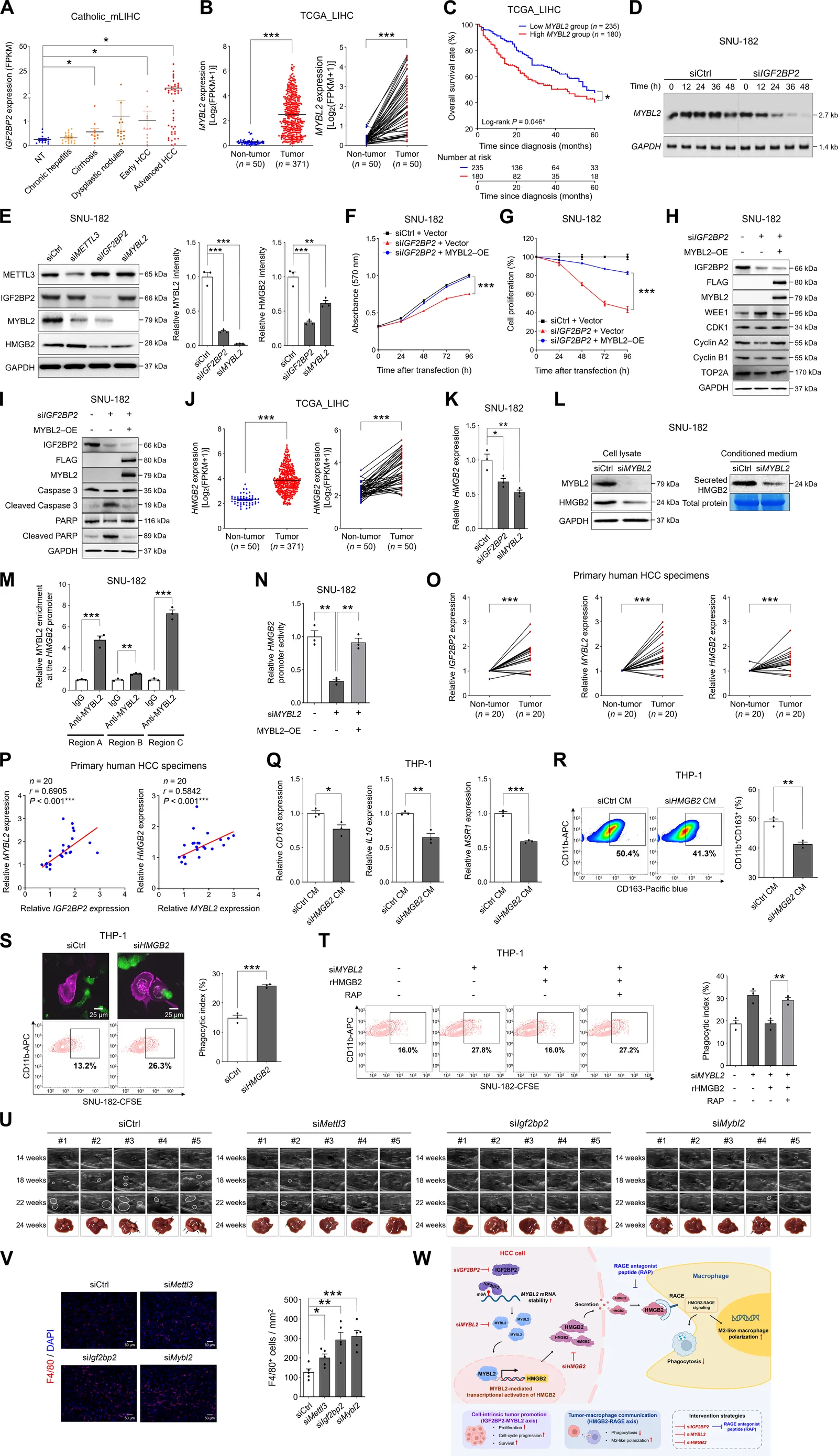
IGF2BP2–MYBL2–HMGB2 axis regulates macrophage phagocytosis. (A to C) *IGF2BP2* and *MYBL2* are up-regulated and associated with poor prognosis in HCC. (A) *IGF2BP2* mRNA expression across multistage HCC progression in the Catholic_mLIHC cohort (nontumor, *n* = 15; chronic hepatitis, *n* = 20; cirrhosis, *n* = 10; dysplastic nodules, *n* = 17; early HCC, *n* = 11; advanced HCC, *n* = 45). (B) Differential expression of *MYBL2* in the TCGA_LIHC cohort validated by unpaired and paired analyses. (C) Kaplan–Meier analysis of overall survival in TCGA_LIHC patients according to *MYBL2* expression. Patients were classified into high and low expression groups using the median *MYBL2* mRNA expression value of the cohort as the cutoff. (D and E) m6A-dependent IGF2BP2 stabilizes *MYBL2* mRNA in HCC. (D) Northern blot analysis of *MYBL2* RNA levels in SNU-182 cells transfected with siCtrl or si*IGF2BP2* at the indicated time points (0, 12, 24, 36, and 48 h), demonstrating reduced *MYBL2* RNA stability following *IGF2BP2* knockdown. GAPDH was used as a loading control. (E) Western blot analysis showing decreased MYBL2 and HMGB2 protein expression in SNU-182 cells following *METTL3*, *IGF2BP2*, or *MYBL2* depletion. Densitometric quantification of MYBL2 and HMGB2 protein levels normalized to GAPDH is shown on the right. (F to I) Aberrant MYBL2 expression contributes to hepatocarcinogenesis. (F) MTT assay demonstrating reduced cell growth following *IGF2BP2* knockdown and restoration of proliferative capacity by ectopic MYBL2 expression (MYBL2–OE) in SNU-182 cells. (G) BrdU incorporation assay evaluating proliferative activity under the indicated conditions. (H) Western blot analysis of cell-cycle regulatory proteins following modulation of IGF2BP2 and MYBL2 expression. FLAG denotes the FLAG (DYK) epitope tag fused to the MYBL2 overexpression construct and was used to verify ectopic MYBL2 expression. (I) Western blot analysis of apoptosis-associated markers. These results indicate that aberrant MYBL2 expression contributes to hepatocarcinogenesis and functions as a critical downstream effector of IGF2BP2. (J to N) Clinical relevance of HMGB2 and transcriptional activation of HMGB2 by MYBL2 in HCC. (J) *HMGB2* mRNA expression in the TCGA_LIHC cohort validated by unpaired and paired analyses. (K) *HMGB2* mRNA expression in SNU-182 cells transfected with siCtrl, si*IGF2BP2*, or si*MYBL2*, measured by qRT-PCR. (L) Intracellular and secreted HMGB2 protein levels following *MYBL2* knockdown, with Coomassie blue staining used as a loading control for conditioned medium in SNU-182 cells. (M) ChIP–qPCR analysis demonstrating enrichment of MYBL2 binding across the indicated *HMGB2* promoter regions. (N) Dual-luciferase reporter assay in SNU-182 cells cotransfected with *HMGB2* promoter constructs and either si*MYBL2* or pcDNA3.1_MYBL2. Luciferase activity was normalized to Renilla activity. Collectively, these findings demonstrate that MYBL2 transcriptionally activates HMGB2 expression and promotes extracellular secretion of HMGB2 in HCC cells. (O and P) Validation of the IGF2BP2–MYBL2–HMGB2 axis in primary human HCC tissues. (O) Relative mRNA expression of *IGF2BP2*, *MYBL2*, and *HMGB2* in 20 pairs of primary human HCC tissues and matched adjacent noncancerous liver tissues, as determined by qRT-PCR. Lines connect matched tissues obtained from the same patient. (P) Correlation analyses of *IGF2BP2* and *MYBL2* mRNA expression and of *MYBL2* and *HMGB2* mRNA expression in primary human HCC tissues. Each dot represents one HCC specimen. These results support the coordinated expression of the IGF2BP2–MYBL2–HMGB2 axis in primary human HCC tissues. (Q to T) HMGB2–RAGE signaling suppresses macrophage-mediated phagocytosis and promotes M2-like macrophage polarization in HCC. (Q) THP-1-derived M0 macrophages were cultured with conditioned medium (CM) from HCC cells transfected with siCtrl or si*HMGB2*, and M2-associated macrophage gene expression was analyzed by qRT-PCR. (R) Flow cytometric analysis of M2-like polarization in THP-1-derived M0 macrophages cultured with conditioned medium from SNU-182 cells transfected with siCtrl or si*HMGB2*. Representative density plots (left) and quantification of CD11b^+^CD163^+^ cells (right) are shown. (S) Phagocytosis of SNU-182 cells by M1-polarized THP-1-derived macrophages following *HMGB2* knockdown. Representative fluorescence images (top) and flow cytometric quantification of phagocytic uptake (bottom) are shown. Increased phagocytic uptake was observed without induction of tumor-cell apoptosis, indicating that the phenotype was not primarily attributable to an increased apoptotic burden. (T) Flow cytometric analysis of macrophage phagocytosis following si*MYBL2* transfection, with modulation by recombinant HMGB2 (rHMGB2; 500 ng) and the RAGE antagonist peptide (RAP; 10 μM) during macrophage–tumor cell coculture. Recombinant HMGB2 attenuated the increased phagocytosis induced by *MYBL2* depletion, whereas blockade of HMGB2–RAGE interaction by RAP restored macrophage phagocytic activity, demonstrating that tumor-derived HMGB2 suppresses macrophage-mediated tumor-cell clearance through RAGE-dependent signaling. (U and V) In vivo validation of the IGF2BP2–MYBL2–HMGB2 regulatory axis in hepatocarcinogenesis. (U) Representative ultrasonography images of H-*ras* transgenic mice treated with siCtrl, si*Mettl3*, si*Igf2bp2*, or si*Mybl2* at 14, 18, and 22 weeks of age, followed by representative liver images at 24 weeks of age. White circles indicate hepatic tumor masses identified by ultrasonography, whereas arrows indicate grossly visible hepatic tumors in the livers. These images demonstrate a markedly reduced hepatic tumor burden following inhibition of the IGF2BP2–MYBL2 pathway. (V) Representative F4/80 immunofluorescence images of liver tumor tissues from H-*ras* transgenic mice treated with siCtrl, si*Mettl3*, si*Igf2bp2*, or si*Mybl2*. F4/80-positive macrophages are shown in red, and nuclei were counterstained with DAPI (blue). Macrophage density was quantified as the number of F4/80-positive cells per square millimeter of tumor tissue. These findings indicate that disruption of the IGF2BP2–MYBL2–HMGB2 pathway is associated with increased macrophage activity within the tumor microenvironment. (W) Schematic model of the proposed IGF2BP2–MYBL2–HMGB2 regulatory axis. IGF2BP2 recognizes and stabilizes m6A-modified *MYBL2* mRNA, resulting in increased MYBL2 expression. MYBL2 transcriptionally activates HMGB2, and secreted HMGB2 engages RAGE on macrophages, promotes an M2-like macrophage phenotype, and suppresses macrophage-mediated phagocytosis. The model also illustrates the tumor-cell-intrinsic functions of the IGF2BP2–MYBL2 axis and the genetic and pharmacological interventions used to disrupt the pathway. Quantitative data are presented as the mean ± SEM. Statistical significance was determined using 2-tailed paired or unpaired Student’s *t* tests, one-way ANOVA with the indicated post hoc test, the log-rank test, or Pearson’s correlation analysis, as appropriate. *P < 0.05, **P < 0.01, and ***P < 0.001. ANOVA, analysis of variance; APC, allophycocyanin; BrdU, 5-bromo-2′-deoxyuridine; CD11b, cluster of differentiation 11b; CD163, cluster of differentiation 163; CDK1, cyclin-dependent kinase 1; CFSE, carboxyfluorescein succinimidyl ester; ChIP, chromatin immunoprecipitation; CM, conditioned medium; DAPI, 4′,6-diamidino-2-phenylindole; FPKM, fragments per kilobase of transcript per million mapped reads; GAPDH, glyceraldehyde-3-phosphate dehydrogenase; HCC, hepatocellular carcinoma; *HMGB2*, high-mobility group box 2; *IGF2BP2*, insulin-like growth factor 2 mRNA-binding protein 2; IgG, immunoglobulin G; IL10, interleukin 10; LIHC, liver hepatocellular carcinoma; m6A, N6-methyladenosine; *METTL3*, methyltransferase-like 3; MSR1, macrophage scavenger receptor 1; MTT, 3-(4,5-dimethylthiazol-2-yl)-2,5-diphenyltetrazolium bromide; *MYBL2*, MYB proto-oncogene like 2; OE, overexpression; PARP, poly(ADP-ribose) polymerase; qPCR, quantitative polymerase chain reaction; qRT-PCR, quantitative reverse transcription polymerase chain reaction; RAGE, receptor for advanced glycation end products; RAP, RAGE antagonist peptide; rHMGB2, recombinant HMGB2; SEM, standard error of the mean; siRNA, small interfering RNA; TCGA, The Cancer Genome Atlas; TOP2A, DNA topoisomerase IIα; WEE1, WEE1 G2 checkpoint kinase.

*IGF2BP2* mRNA expression was positively correlated with *MYBL2* mRNA expression (Fig. S2A). HCC cell lines SNU-182 and Hep3B exhibited relatively high *MYBL2* mRNA expression compared with MIHA, an immortalized nontransformed hepatocyte cell line (Fig. S2B and C). *IGF2BP2* knockdown progressively reduced *MYBL2* mRNA expression (Fig. S2D) and shortened its half-life (Fig. S2E). Consistently, Northern blot analysis confirmed that IGF2BP2 regulates the stability of *MYBL2* mRNA (Fig. 1D). RIP assays indicated that methyltransferase-like 3 (METTL3), IGF2BP2, and m6A were enriched on *MYBL2* mRNA (Fig. S2F), and *METTL3* knockdown reduced IGF2BP2 binding to *MYBL2* mRNA (Fig. S2G). Accordingly, knockdown of *IGF2BP2* significantly decreased MYBL2 protein expression (Fig. 1E and Fig. S2H). Together, these findings indicate that IGF2BP2 stabilizes m6A-modified *MYBL2* mRNA, enhancing MYBL2 protein expression in HCC. Furthermore, *MYBL2* depletion significantly suppressed tumorigenic phenotypes, whereas ectopic MYBL2 expression restored the impaired proliferative capacity induced by *IGF2BP2* knockdown (Fig. 1F to I and Figs. S3 and S4).

To identify MYBL2 downstream targets, we analyzed a MYBL2 chromatin immunoprecipitation sequencing (ChIP-seq) dataset and identified high mobility group box 2 (*HMGB2*) as the target with the strongest MYBL2 binding enrichment (Fig. S5A and Table S3). *HMGB2* mRNA was up-regulated in HCC and associated with advanced disease stage and worse overall survival (Fig. 1J and Fig. S5B to E). *HMGB2* depletion also suppressed tumorigenic phenotypes in HCC (Fig. S5F to H).

*HMGB2* mRNA expression exhibited a significant positive correlation with *MYBL2* mRNA expression (Fig. S6A). Consistent with these findings, knockdown of either *IGF2BP2* or *MYBL2* significantly reduced *HMGB2* mRNA expression (Fig. 1K and Fig. S6B). Western blot analysis further showed that depletion of either *IGF2BP2* or *MYBL2* decreased HMGB2 protein levels (Fig. 1E and Fig. S2H). Given previous evidence that HMGB2 can translocate to the cytosol and subsequently be secreted into the extracellular space [5], *MYBL2* depletion also decreased secreted HMGB2 protein levels (Fig. 1L and Fig. S6C). Chromatin immunoprecipitation followed by quantitative polymerase chain reaction (ChIP–qPCR) analysis revealed significant enrichment of MYBL2 at 3 selected regions of the *HMGB2* promoter containing putative MYBL2 binding sites (Fig. 1M and Fig. S6D). *MYBL2* knockdown significantly reduced *HMGB2* promoter-driven luciferase activity, while MYBL2 overexpression rescued this effect and restored promoter activity (Fig. 1N and Fig. S6E). These results indicate that MYBL2 acts as a functional transcriptional activator of HMGB2. To assess the clinical relevance of this regulatory axis, we analyzed 20 randomly selected pairs of primary human HCC tissues and matched adjacent noncancerous liver tissues. Among these, 16 pairs (80%) showed concordant up-regulation of *IGF2BP2*, *MYBL2*, and *HMGB2* in HCC tissues (Fig. 1O and Fig. S6F). Their mRNA expression levels were positively correlated (Fig. 1P), and Western blot analysis further demonstrated concordant increases in the corresponding protein levels (Fig. S6G).

HMGB2 is a close paralog of high mobility group box 1 (HMGB1), sharing conserved HMG-box domains and high sequence similarity [5,6]. A recent multi-omics study linked HMGB2 to an immunosuppressive tumor microenvironment in HCC [7], while immune cell deconvolution analyses revealed macrophages as the predominant immune cell population in HCC (Fig. S7A). Because tumor-derived HMGB1 promotes M2-like macrophage polarization [8], we investigated whether HMGB2 exerts similar immunomodulatory effects. Macrophages cultured in conditioned medium from HMGB2-depleted HCC cells exhibited reduced M2-associated marker expression (Fig. 1Q). Consistently, flow cytometric analysis showed a decrease in the proportion of cells positive for both cluster of differentiation 11b (CD11b^+^) and cluster of differentiation 163 (CD163^+^) (Fig. 1R). To determine whether this phenotypic shift was accompanied by functional changes in macrophage effector activity, we next quantified macrophage phagocytosis. *HMGB2* silencing significantly increased macrophage phagocytic uptake (Fig. 1S and Fig. S7B). Importantly, *HMGB2* depletion increased macrophage uptake independently of tumor-cell apoptosis (Fig. S7C). To further substantiate that HMGB2 regulates macrophage phagocytosis, we investigated the potential involvement of receptor for advanced glycation end products (RAGE), given prior reports that HMGB2 can bind to RAGE [9,10]. Coimmunoprecipitation assays demonstrated that HMGB2 interacts with macrophage-expressed RAGE (Fig. S7D). As MYBL2 transcriptionally regulates HMGB2, *MYBL2* knockdown increased macrophage phagocytosis. However, this increase was abrogated by treatment with recombinant HMGB2 (rHMGB2). Notably, blockade of RAGE using the RAGE antagonist peptide (RAP), which prevents HMGB2 binding to RAGE, restored the enhanced phagocytosis even in the presence of rHMGB2, indicating that HMGB2 restrains macrophage phagocytosis through RAGE-dependent signaling (Fig. 1T and Fig. S7E). Consistent with the upstream role of IGF2BP2 in this axis, *IGF2BP2* knockdown also increased macrophage-mediated phagocytosis, and this increase was attenuated by rHMGB2 treatment. Although increased efferocytosis may contribute to the enhanced macrophage uptake observed following *IGF2BP2* or *MYBL2* depletion, the ability of *HMGB2* depletion and rHMGB2 rescue to modulate phagocytosis independently of apoptosis indicates that HMGB2 is a key downstream regulator of tumor–macrophage communication (Fig. S7F). To determine whether this phagocytic phenotype was restricted to THP-1-derived macrophages, we further performed phagocytosis assays using mouse primary peritoneal macrophages. Depletion of *Mybl2* or *Hmgb2* in mouse HCC cells significantly increased their phagocytosis by mouse primary peritoneal macrophages compared with siCtrl-transfected cells, consistent with the increased phagocytic uptake observed in THP-1-derived macrophages (Fig. S7G). Furthermore, consistent findings were obtained in a cross-species coculture experiment using mouse primary peritoneal macrophages and human HCC cells (Fig. S7H).

Next, to demonstrate the in vivo relevance of IGF2BP2, MYBL2, and HMGB2 in liver tumorigenesis, we employed H-*ras* transgenic mice that spontaneously develop liver cancer. Mice treated with si*Mettl3*, si*Igf2bp2*, or si*Mybl2* exhibited a markedly reduced tumor burden, with fewer and smaller hepatic tumors and a lower tumor incidence (Fig. 1U and Fig. S8A and B). Consistently, the liver weight-to-body weight (LW/BW) ratio analysis showed that depletion of *Mettl3*, *Igf2bp2*, or *Mybl2* shifted the tumor-burden classification from the mild tumor-burden range observed in the siCtrl group to the normal-liver range, further supporting an overall reduction in tumor burden (Fig. S8C and Table S4). Notably, Hmgb2 protein levels were markedly reduced following *Mybl2* knockdown, supporting Hmgb2 as a downstream effector of Mybl2 in liver tissues (Fig. S8D and E). F4/80 immunofluorescence analysis further revealed increased macrophage activity in liver tumor tissues from mice treated with si*Mettl3*, si*Igf2bp2*, or si*Mybl2* compared with the siCtrl group (Fig. 1V). Similarly, RAP or si*Hmgb2* treatment increased F4/80-positive macrophage activity in liver tumor tissues from H-*ras* transgenic mice compared with the corresponding control groups (Fig. S8F).

In summary, our study delineates an IGF2BP2–MYBL2–HMGB2 regulatory axis in HCC, whereby IGF2BP2 stabilizes *MYBL2* mRNA, promoting *HMGB2* transcription. IGF2BP2 and MYBL2 support tumor growth through their established tumor-intrinsic functions, whereas HMGB2 facilitates immune evasion by suppressing macrophage phagocytosis. Thus, reduced tumor proliferation following *IGF2BP2* or *MYBL2* depletion and enhanced phagocytosis following *HMGB2* depletion represent parallel consequences of disrupting this axis (Fig. 1W). These findings link posttranscriptional stabilization of MYBL2 to macrophage-dependent tumor clearance and identify this pathway as a potential therapeutic target in HCC, although its translational relevance requires further validation.

## Ethical Approval

This study analyzed publicly available and previously established de-identified human transcriptomic datasets. The use of institutional human data was approved by the Institutional Review Board of The Catholic University of Korea, College of Medicine (approval numbers: MC22TESI0069 and MC25TESI0098). Written informed consent was obtained from all participants. All animal experiments involving H-*ras* transgenic mice were reviewed and approved by the Institutional Animal Care and Use Committee of The Catholic University of Korea, College of Medicine (approval number: 2025-0062-08). Animal care and experimental procedures were performed in accordance with the institutional guidelines for the care and use of laboratory animals.

## Data Availability

The publicly available datasets analyzed in this study were obtained from The Cancer Genome Atlas (TCGA), including the Liver Hepatocellular Carcinoma (TCGA_LIHC) cohort and other TCGA cancer cohorts used for pan-cancer analyses, the International Cancer Genome Consortium Liver Cancer–RIKEN, Japan (ICGC_LIRI), and the Gene Expression Omnibus (GEO) database of the National Center for Biotechnology Information (NCBI). The GEO accession numbers used in this study are GSE90639, GSE90684, GSE170799, GSE77314, GSE101685, and GSE114564, as described in Supplementary Materials and Methods. Other data supporting the findings of this study are available from the corresponding author upon reasonable request.
