# Supplementary material for: An IGF2BP2–MYBL2–HMGB2 Signaling Axis Promotes Macrophage Immune Evasion in Hepatocellular Carcinoma
Source: Cancer Commun (Lond). 2026 Sep 30;46:0058. doi: 10.34133/cancomm.0058 (PMC13624569; doi:10.34133/cancomm.0058)
Supplement: Supplementary file 1 [file cancomm.0058.f1.docx]

**Supplementary Materials for**

**An IGF2BP2–MYBL2–HMGB2 Signaling Axis Promotes Macrophage Immune Evasion in Hepatocellular Carcinoma**

Jin Woong Ha^1,2,3^, Sang Yean Kim^1,2,5^, Soyoung Jeon^1,2,3^, Min Jeong Na^1,2,5^, Jung Hwan Yoon^1,2,4^, and Suk Woo Nam^1,2,3,5*^

^1^Department of Pathology, College of Medicine, The Catholic University of Korea, Seoul, Republic of Korea.

^2^Functional RNomics Research Center, The Catholic University of Korea, Seoul, Republic of Korea.

^3^Department of Biomedicine & Health Sciences, Graduate School, The Catholic University of Korea, Seoul, Republic of Korea.

^4^Institute of Hansen's Disease, College of Medicine, The Catholic University of Korea, Seoul, Republic of Korea.

^5^NEORNAT Inc., Seoul, Republic of Korea

^*^Address Correspondence to: S.W.N (Suk Woo Nam), swnam@catholic.ac.kr

**Supplementary Materials and Methods**

**Phagocytosis assay**

At 48 h post-transfection, cells were detached using Accutase, washed with phosphate-buffered saline (PBS), and labeled with carboxyfluorescein succinimidyl ester (CFSE; Cat. No. C34554, Thermo Fisher Scientific) (2 μmol/L, 15 min). THP-1 monocytes were differentiated into macrophages with 100 ng/mL phorbol 12-myristate 13-acetate (PMA; Cat. No. P1585, Sigma-Aldrich) and polarized toward an M1 phenotype using 100 ng/mL lipopolysaccharide (LPS; Cat. No. L2630, Sigma-Aldrich) and 20 ng/mL interferon gamma (IFN-γ; Cat. No. 300-02, PeproTech). Thioglycollate-elicited peritoneal macrophages were isolated 3 days after thioglycollate injection and polarized toward an M1 phenotype. CFSE-labeled cells were co-cultured with macrophages at an effector-to-target ratio of 1:2 for 2 h at 37°C. Where indicated, recombinant high mobility group box 2 (HMGB2) was added at 500 ng, or the receptor for advanced glycation end products (RAGE) antagonist peptide was added at 10 μmol/L. Cells were harvested and analyzed by FACS Canto^TM^ flow cytometer (BD Biosciences). Debris and doublets were excluded based on forward and side scatter properties, and macrophages were gated on the CD11b⁺ population. Phagocytosis was quantified as the percentage of CFSE⁺ cells within the CD11b⁺ macrophage gate (**Figure S7I and J**). For experiments using primary mouse peritoneal macrophages, phagocytosis was quantified as the percentage of CFSE^+^ cells within the F4/80^+^ macrophage gate.

**Flow cytometry analysis of M2-like macrophage polarization**

THP-1 monocytes were differentiated into M0 macrophages with 100 ng/mL PMA (Cat. No. P1585, Sigma-Aldrich). After differentiation, cells were incubated with conditioned medium collected from cancer cells at a final concentration of 50% for 24 h to induce macrophage polarization. Macrophages were harvested, washed with PBS containing 1% fetal bovine serum (FBS; Cat. No. F0900-050, GenDEPOT), and stained with a Pacific Blue-conjugated anti-CD163 antibody (Cat. No. 333611, BioLegend) to assess M2-like polarization by the FACS Canto^TM^ flow cytometer (BD Biosciences). For analysis, debris and doublets were excluded based on forward and side scatter properties, and the proportion of CD163-positive cells within the CD11b-positive macrophage population was quantified.

***In vivo* tumorigenesis and intervention studies**

Male H-*ras*-transgenic mice were used as a spontaneous liver tumor model [1, 2]. Beginning at 14 weeks of age, mice were assigned to the siCtrl, si*Mettl3*, si*Igf2bp2*, or si*Mybl2* treatment groups. The indicated siRNAs were formulated with Invivofectamine 3.0 (Cat. No. IVF3005, Invitrogen) and administered intravenously via tail-vein injection at 0.25 mg/kg once weekly from 14 to 23 weeks of age, for a total of 10 injections. The ultrasonography images were obtained at 14, 16, 18, 20 and 22 weeks of age using an ultrasound system (Philips) by the same medical imaging expert each time. The final siRNA injection was administered at 23 weeks of age, and final ultrasonographic assessment was performed at 24 weeks of age. Mice were subsequently sacrificed at 24 weeks of age, and the livers were collected for further analysis. For the additional HMGB2–RAGE intervention study, age-matched male H-*ras* transgenic mice with ultrasonographically detectable hepatic tumor masses were randomly assigned to the control, si*Hmgb2*, or RAGE antagonist peptide (RAP) group. Invivofectamine 3.0 formulated si*Hmgb2* was administered intravenously, whereas RAP (Cat. No. 553031, Sigma-Aldrich) was administered intraperitoneally at 100 μg per mouse once daily. All mice were sacrificed on day 7, and their livers were collected.

**Cell culture**

Human hepatocellular carcinoma (HCC) cell lines (Hep3B, HepG2, Huh7, PLC/PRF/5, SNU-182, SNU-354, SNU-368, SNU-387, SNU-423, SNU-449, SNU-475, SNU-739, SNU-761, SNU-878, and SNU-886) were obtained from Korean Cell Line Bank (KCLB). MIHA immortalized hepatocyte cells were kindly provided by Dr. Jayanta Roy-Chowdhury (Albert Einstein College of Medicine). Each cell line was cultured in EMEM (ATCC), RPMI-1640 or DMEM medium (GenDEPOT) containing 10% fetal bovine serum (FBS; GenDEPOT) and 100 units/mL of penicillin-streptomycin (GenDEPOT). All cells were cultured at 37℃ in a humidified incubator with 5% CO_2_.

**Transfection**

Small interfering RNAs (siRNAs) were synthesized by Genolution or Macrogen. The sequences of the siRNAs are listed in **Table S5**. Human MYB proto-oncogene-like 2 (MYBL2) protein expression plasmid, subcloning gene ORF sequence (MYBL2: NM_002466.4) in pcDNA3.1+/C-(K)-DYK plasmid, was purchased from Genscript^TM^. Transfections were carried out using Lipofectamine RNAiMAX (Cat. No. 13778150, Invitrogen) or Lipofectamine 2000 reagent (Cat. No. 11668019, Invitrogen), according to the manufacturer’s instructions.

**RNA extraction and quantitative real-time polymerase chain reaction (qRT-PCR)**

Total RNA was isolated from cell lines and frozen human tissues using TRIzol reagent (Cat. No. 15596018, Invitrogen) according to the manufacturer's instructions. For synthesis of cDNAs specific to mRNAs, Tetro cDNA synthesis kit (Cat. No. BIO-65043, Bioline) was used. qRT-PCR was performed with a GoTaq® qPCR Master Mix (Cat. No. A6002, Promega). To normalize differences in the amount of total cDNA added to each reaction, glyceraldehyde-3-phosphate dehydrogenase (GAPDH) was used as the endogenous control for mRNA expression.

**Western blotting analysis**

Mouse HCC tissues and human HCC cell lines were lysed in protein extraction buffer (50 mmol/L of HEPES, 5 mmol/L of ethylenediaminetetraacetic acid, 50 mmol/L of NaCl, 1% Triton X-100, 50 mmol/L of NaF, 10 mmol/L of Na_2_P_2_O_7_, 1 mmol/L of Na_3_VO_4_, and 100X Halt protease inhibitor cocktail or 100X Halt protease & phosphatase inhibitor). Lysates containing equal amounts of proteins were separated by sodium dodecyl sulfate/polyacrylamide gel electrophoresis and transferred onto polyvinylidene difluoride membrane (Cat. No. 1620177, Bio-Rad). The membranes were blocked with 5% skim milk and incubated with the indicated primary antibodies. Antibody information is summarized in **Table S6**. The Immobilon western detection system (Cat. No. WBKLS0050, Merck Millipore) was used to detect antibodies. The intensities of the western blot bands were quantified using iBright™ CL1500 Imaging System (Thermo Fisher Scientific).

**Cell growth assay**

Cells were seeded in a 12-well plate to 40% confluency for transfection. After transfection, cells were incubated with 0.5 mg/mL of MTT [3-(4,5-dimethylthiazol-2-yl)-2,5-diphenyltetrazolium bromide] solution (Cat. No. MC1029-001-02, Biosesang) for 1 h. The dark blue formazan products formed by viable cells were dissolved in dimethyl sulfoxide (DMSO; Cat. No. D2650, Sigma-Aldrich), and absorbance was measured using a Varioskan™ LUX Multimode Microplate Reader (Thermo Fisher Scientific).

**5-Bromo-2’-deoxyuridine (BrdU) incorporation assay**

Cells were seeded in a 24-well plate to 40% confluency for transfection. After transfection, cells were incubated with BrdU reagent for 4 h. The assay was performed every 24 h according to a BrdU cell proliferation assay kit (Millipore) manufacturer’s protocol. The final product was quantified at 450 nm by Varioskan™ LUX Multimode Microplate Reader.

**Clonogenic assay**

Cells were transfected with siRNA in 60-mm cell culture dishes. After transfection for 24 h, the cells were reseeded in 6-well plates and incubated for 2 weeks. Next, the cells were washed with PBS and fixed with 1% paraformaldehyde for 30 min at room temperature. The fixed cells were stained with 0.5% crystal violet for 1 h at room temperature. Colonies were counted using a clono-counter program.

**Cell cycle analysis**

Cells were transfected with siRNAs in 60-mm cell culture dishes. After 48 h of incubation, the cells were harvested by Trypsin-EDTA, washed with cold PBS, fixed in 70% ethanol, resuspended in 200 μL PBS with 1 mg/mL RNase A (Cat. No. R4642, Sigma-Aldrich), 50 μg/mL propidium iodide (Cat. No. P4864, Sigma-Aldrich) and 1% Triton X-100. Cells were incubated in the dark for 45 min at 37℃. Stained cells fractions were detected using FACS Canto^TM^ flow cytometer (BD Biosciences).

**Apoptosis analysis**

The Annexin V-FITC Apoptosis Detection Kit I (Cat. No. 556419, BD Biosciences) was used to measure the level of apoptosis. After transfection for 48 h, cells were harvested by Trypsin-EDTA, washed with PBS, and resuspended using 1× binding buffer. Then 100 μL containing 1 × 10^5^ cells were transferred to a 5 mL culture tube and mixed with 5 μL of Annexin V-FITC and 10 μL of propidium iodide (Cat. No. P4864, Sigma-Aldrich) solution. After 15 min at room temperature in the dark, 400 μL of 1× binding buffer was added to each tube, and apoptotic fractions were detected using the FACS Canto^TM^ flow cytometer (BD Biosciences).

**RNA stability assay**

Cells were transfected with siRNAs in 60-mm cell culture dishes. After 48 h of incubation, transcription was inhibited by treatment with 10 μg/mL actinomycin D (Cat. No. A9415, Sigma-Aldrich). Total RNA was harvested at the indicated time points after actinomycin D addition and extracted using TRIzol reagent (Cat. No. 15596018, Invitrogen) according to the manufacturer’s instructions. For the synthesis of cDNAs specific to mRNAs, the Tetro cDNA synthesis kit (Cat. No. BIO-65043, Bioline) was used, and mRNA abundance was quantified by qRT-PCR using GoTaq® qPCR Master Mix (Cat. No. A6002, Promega). Transcript levels were normalized to GAPDH.

**RNA immunoprecipitation (RIP) assay**

RIP assays were performed using a RIP assay kit (Cat. No. RN1001, MBL) in accordance with the manufacturer’s protocol. Briefly, HCC cell lines were lysed, and 10% of the lysate was reserved for total RNA isolation. The clarified cell lysates were incubated overnight at 4°C with 15 μg of antibody. RNA was extracted on the beads and amplified with RT-PCR and qRT-PCR. Primer sequence information is summarized in **Table S7**.

**Chromatin immunoprecipitation assay**

Chromatin immunoprecipitation (ChIP) assay was performed using the Pierce Agarose ChIP Kit (Cat. No. 26156, Thermo Fisher Scientific) according to the manufacturer’s instructions. Immunoprecipitation was carried out using an anti-MYBL2 antibody to enrich MYBL2-bound chromatin, alongside the kit-recommended control IgG. The *HMGB2* promoter was examined for putative MYBL2 binding sites, and three promoter regions (regions A, B, and C) encompassing these sites were selected for ChIP–qPCR analysis. Specific primer pairs were designed to amplify each region, and the corresponding primer sequences are provided in **Table S7**.

**Publicly available genomic data analyses**

To investigate the expression levels of candidate genes in HCC, transcriptomic data were retrieved from multiple independent cohorts: The Cancer Genome Atlas Liver Hepatocellular Carcinoma (TCGA_LIHC), the International Cancer Genome Consortium Liver Cancer–RIKEN, Japan (ICGC_LIRI), and the Gene Expression Omnibus (GEO) database of the National Center for Biotechnology Information (NCBI). The Catholic_mLIHC cohort corresponds to the publicly available RNA-sequencing dataset GSE114564 and was used to evaluate gene expression across multistage liver disease. The GEO accession numbers used in this study were GSE90639, GSE90684, GSE170799, GSE77314, GSE101685, and GSE114564. Level 3 HTSeq-FPKM mRNA expression data from TCGA datasets were log2-transformed [log2(FPKM+1)] and used to assess gene expression levels.

**Mouse liver cancer model**

The H-*ras*12V homozygous transgenic (*Ras*-Tg) mice were kindly provided by Dr. Dae-Yeoul Yu (Laboratory of Human Genomics, Korea Research Institute of Bioscience and Biotechnology) [1]. Male mice spontaneously developed liver cancer beginning at approximately 15-18 weeks of age. Mouse livers were harvested at 24 weeks of age and processed for experiments. All animal research procedures were performed in accordance with the Laboratory Animals Welfare Act, the Guide for Care and Use of Laboratory Animals, and the Guidelines and Policies for Rodent Experiment provided by the IACUC (Institutional Animal Care and Use Committee) at the Catholic University of Korea College of Medicine.

**Northern blotting**

Twenty micrograms of total RNA and four micrograms of RNA marker (Cat. No. G3191, Promega) were separated by electrophoresis on 1% agarose-formaldehyde-MOPS gels. The RNA was transferred to a Biodyne Transfer Membrane (Pall corporation) via capillary blotting in 20× SSC for 16 h. RNA was cross-linked to the biodyne membrane by UV crosslinker for 1 min. The membrane was incubated in a rotating oven at 65°C in 10 mL hybridization buffer for 30 min without radioactive probe. Single-strand probes were generated by the incorporation of radioactive dATP into cDNA using a unidirectional thermocycling ratio. The probe purification was performed using Microspin G-50 columns (Cat. No. 27533001, Cytiva). The probe was added to 10 mL hybridization buffer in an amount of 1-5 million CPM/mL and incubated overnight at 65°C in a rotating oven. The membrane was washed with wash buffer Ⅰ (2× SSC, 0.1% SDS) and wash buffer Ⅱ (0.1× SSC, 0.1% SDS) at 65°C for 15 min. A phosphor image analyzer (Bio-Rad) was used for imaging [3].

**Statistical analysis**

Survival curves were plotted using the Kaplan-Meier product limit method, and significant differences between survival curves were determined using the log-rank test. All experiments were performed at least three times, and all samples were analyzed in triplicate. Results are presented as mean ± standard error of the mean (SEM). Statistical differences between groups were evaluated by paired or unpaired two-tailed Student’s *t* test using GraphPad™ 8.4 software. *P* values < 0.05 were considered statistically significant.

**Dual-luciferase reporter assay for HMGB2 promoter activity**

The promoter region of HMGB2 was amplified by PCR and cloned into the pGL4.23 (Cat. No. E8411, Promega) luciferase reporter vector upstream of the firefly luciferase gene. All constructs were verified by Sanger sequencing. Cells were seeded in 12-well plates and co-transfected with the HMGB2 promoter–pGL4.23 construct (200 ng per well) together with either siRNA or expression plasmid. To normalize for transfection efficiency, the pRL-TK (Cat. No. E2241, Promega) Renilla luciferase plasmid (20 ng per well) was co-transfected as an internal control. At 48 h post-transfection, luciferase activity was measured using the Dual-Luciferase Reporter Assay System (Cat. No. E1910, Promega) according to the manufacturer’s instructions. Firefly luciferase activity was normalized to Renilla luciferase activity, and results were expressed as relative luciferase activity compared with the corresponding control group. Each experiment was performed in triplicate wells and repeated independently at least three times.

**FLAG immunoprecipitation**

Cells were transfected with pcDNA3.1_HMGB2 plasmid (FLAG-tagged). At 48 h post-transfection, conditioned medium (CM) was collected and centrifuged to remove cellular debris before use. CM was applied to PMA-differentiated THP-1 macrophages (50% CM diluted in fresh culture medium, v/v) followed by overnight incubation. THP-1–derived macrophages were then crosslinked with 0.5% formaldehyde for 10 min at room temperature, quenched with 125 mmol/L glycine for 5 min, and lysed in protein extraction buffer (50 mmol/L of HEPES, 5 mmol/L of ethylenediaminetetraacetic acid, 50 mmol/L of NaCl, 1% Triton X-100, 50 mmol/L of NaF, 10 mmol/L of Na_2_P_2_O_7_, 1 mmol/L of Na_3_VO_4_, and 100X Halt protease inhibitor cocktail). Lysates were clarified by centrifugation, and 5% of each lysate was reserved as input. Clarified lysates were subjected to FLAG immunoprecipitation using anti-FLAG M2 Magnetic Beads (Cat. No. M8823, Sigma-Aldrich) with overnight rotation at 4°C. Beads were washed, and bound proteins were eluted in SDS sample buffer and analyzed by western blotting. Immunoprecipitates and input lysates were probed with anti-FLAG, anti-HMGB2, and anti-RAGE antibodies (**Table S6**).

**Tissue samples**

A total of 20 pairs of human HCC tissues and matched adjacent non-cancerous liver tissues were obtained from the Biobank of InJe University Paik Hospital (InjeBiobank), a member of Korea Biobank Network. Written informed consent was obtained from all participants, and the study was conducted in accordance with the Declaration of Helsinki. The study protocol was approved by the Institutional Review Board of the Songeui Campus, College of Medicine, The Catholic University of Korea (IRB approval number: MC22TESI0069, MC25TESI0098). The clinicopathological characteristics of the 20 patients, including age, sex, etiology, background liver disease, tumor size and multiplicity, American Joint Committee on Cancer tumor–node–metastasis stage, histological grade, and serum alpha-fetoprotein levels, are summarized in **Table S8**.

**Immunofluorescence**

Frozen liver tumor tissues from H-*ras* transgenic mice were embedded in optimal cutting temperature compound and sectioned using a cryostat. The frozen sections were air-dried and fixed with cold acetone for 10 min. After washing with PBS, the sections were blocked with 5% bovine serum albumin in PBS for 1 h at room temperature. The sections were incubated overnight at 4°C with a directly fluorophore-conjugated anti-F4/80 antibody. After washing with PBS, nuclei were counterstained with 4′,6-diamidino-2-phenylindole (DAPI; Cat. No. P36941, Invitrogen), and the sections were mounted using an antifade mounting medium. Fluorescence images were acquired using an EVOS M5000 Imaging System (Thermo Fisher Scientific). Samples from all experimental groups were processed in parallel and imaged under identical acquisition settings. For each mouse, five randomly selected, non-overlapping microscopic fields were analyzed. F4/80-positive cells were counted, and macrophage density was expressed as the number of F4/80-positive cells per square millimeter of tissue. The values obtained from the five fields were averaged to generate a single mouse-level value, with each mouse considered one biological replicate.

**Supplementary references**

1.	Wang AG, Moon HB, Lee MR, Hwang CY, Kwon KS, Yu SL, Kim YS, Kim M, Kim JM, Kim SK, et al. Gender-dependent hepatic alterations in H-ras12V transgenic mice. J Hepatol. 2005;43(5):836-844.

2.	Kim SY, Ha JW, Na MJ, Jeon S, Yoon JH, Park WS, Nam SW. Oncogenic function of growth arrest-specific transcript 5 by competing with miR-423-3p to regulate SMARCA4 in hepatocellular carcinoma. Exp Mol Med. 2025;57(6):1164-1176.

3.	Marquardt S, Hazelbaker DZ, Buratowski S. Distinct RNA degradation pathways and 3' extensions of yeast non-coding RNA species. Transcription. 2011;2(3):145-154.

**Table S1. Differential expression of m6A regulatory genes across HCC transcriptomic cohorts.**

| Type | No. | Genes | Catholic_mLIHC | | | TCGA_LIHC | | | ICGC_LIRI | | | GSE77314 | | |
| --- | --- | --- | --- | --- | --- | --- | --- | --- | --- | --- | --- | --- | --- | --- |
|  |  |  | Non-tumor | Tumor | Fold change | Non-tumor | Tumor | Fold change | Non-tumor | Tumor | Fold change | Non-tumor | Tumor | Fold change |
|  |  |  | (*n* = 15) | (*n* = 56) |  | (*n* = 50) | (*n* = 371) |  | (*n* = 202) | (*n* = 243) |  | (*n* = 50) | (*n* = 50) |  |
| Writer | 1 | *METTL3* | 12.17 | 14.92 | 1.23 | 1.22 | 2.08 | 1.70 | 11.60 | 19.56 | 1.69 | 19.66 | 28.11 | 1.43 |
|  | 2 | *METTL14* | 18.57 | 15.30 | -1.21 | 1.69 | 1.78 | 1.05 | 5.77 | 5.90 | 1.02 | 7.99 | 5.54 | -1.44 |
|  | 3 | *METTL16* | 4.11 | 4.11 | 1.00 | 0.96 | 1.34 | 1.40 | 1.65 | 2.31 | 1.41 | 3.60 | 3.90 | 1.09 |
|  | 4 | *RBM15* | 8.42 | 9.78 | 1.16 | 1.29 | 1.52 | 1.18 | 2.45 | 2.88 | 1.18 | 1.54 | 1.89 | 1.23 |
|  | 5 | *VIRMA* | 13.72 | 20.43 | 1.49 | 1.59 | 2.44 | 1.54 | 3.86 | 8.60 | 2.22 | 16.15 | 33.90 | 2.10 |
|  | 6 | *WTAP* | 39.04 | 36.28 | -1.08 | 2.57 | 2.94 | 1.15 | 15.51 | 19.91 | 1.28 | 16.90 | 19.82 | 1.17 |
|  | 7 | *CBLL1* | 7.56 | 11.75 | 1.55 | 1.46 | 2.11 | 1.44 | 2.94 | 5.13 | 1.75 | 3.72 | 4.57 | 1.23 |
|  | 8 | *ZC3H13* | 32.36 | 24.35 | -1.33 | 2.11 | 2.11 | 1.00 | 12.56 | 11.29 | -1.11 | 24.87 | 7.60 | -3.27 |
| Eraser | 1 | *ALKBH5* | 23.57 | 18.23 | -1.29 | 4.28 | 4.51 | 1.05 | 12.28 | 15.25 | 1.24 | 16.94 | 15.76 | -1.08 |
|  | 2 | *FTO* | 18.86 | 17.25 | -1.09 | 1.23 | 1.48 | 1.20 | 3.62 | 4.68 | 1.29 | 4.46 | 3.69 | -1.21 |
| Reader | 1 | *EIF3A* | 46.33 | 47.32 | 1.02 | 4.02 | 4.37 | 1.09 | 24.48 | 33.30 | 1.36 | 19.49 | 27.70 | 1.42 |
|  | 2 | *IGF2BP1* | 0.01 | 2.67 | 274.97 | 0.01 | 0.97 | 105.76 | 0.05 | 2.80 | 56.85 | 0.04 | 3.08 | 86.66 |
|  | 3 | *IGF2BP2* | 1.86 | 9.26 | 4.98 | 0.47 | 2.23 | 4.80 | 1.66 | 6.18 | 3.72 | 2.22 | 10.58 | 4.77 |
|  | 4 | *IGF2BP3* | 0.25 | 6.48 | 25.49 | 0.01 | 0.42 | 32.89 | 0.29 | 3.38 | 11.56 | 0.30 | 3.89 | 12.92 |
|  | 5 | *YTHDC1* | NA | NA | NA | 2.37 | 2.63 | 1.11 | 7.66 | 8.20 | 1.07 | NA | NA | NA |
|  | 6 | *YTHDC2* | 15.48 | 15.46 | -1.00 | 1.34 | 1.59 | 1.19 | 5.75 | 6.67 | 1.16 | 7.54 | 5.22 | -1.44 |
|  | 7 | *YTHDF1* | 11.56 | 11.95 | 1.03 | 3.30 | 3.96 | 1.20 | 10.28 | 14.70 | 1.43 | 9.15 | 11.87 | 1.30 |
|  | 8 | *YTHDF2* | 21.73 | 20.54 | -1.06 | 3.23 | 3.62 | 1.12 | 15.10 | 18.14 | 1.20 | 11.55 | 14.15 | 1.22 |
|  | 9 | *YTHDF3* | 25.94 | 28.98 | 1.12 | 3.01 | 3.43 | 1.14 | 10.56 | 16.38 | 1.55 | 8.12 | 8.43 | 1.04 |

Abbreviations: *ALKBH5*, alkB homolog 5, RNA demethylase; *CBLL1*, Cbl proto-oncogene like 1; Catholic_mLIHC, Catholic University multistage liver hepatocellular carcinoma cohort; *EIF3A*, eukaryotic translation initiation factor 3 subunit A; *FTO*, FTO alpha-ketoglutarate dependent dioxygenase; ICGC_LIRI, International Cancer Genome Consortium Liver Cancer–RIKEN, Japan cohort; *IGF2BP1*, insulin like growth factor 2 mRNA binding protein 1; *IGF2BP2*, insulin like growth factor 2 mRNA binding protein 2; *IGF2BP3*, insulin like growth factor 2 mRNA binding protein 3; *METTL3*, methyltransferase 3, N6-adenosine-methyltransferase complex catalytic subunit; *METTL14*, methyltransferase 14, N6-adenosine-methyltransferase non-catalytic subunit; *METTL16*, methyltransferase 16, RNA N6-adenosine; NA, not applicable; *RBM15*, RNA binding motif protein 15; TCGA_LIHC, The Cancer Genome Atlas Liver Hepatocellular Carcinoma; *VIRMA*, vir like m6A methyltransferase associated; *WTAP*, WT1 associated protein; *YTHDC1*, YTH N6-methyladenosine RNA binding protein C1; *YTHDC2*, YTH N6-methyladenosine RNA binding protein C2; *YTHDF1*, YTH N6-methyladenosine RNA binding protein F1; *YTHDF2*, YTH N6-methyladenosine RNA binding protein F2; *YTHDF3*, YTH N6-methyladenosine RNA binding protein F3; *ZC3H13*, zinc finger CCCH-type containing 13.

**Table S2. Differential expression of IGF2BP2-associated candidate target genes in TCGA_LIHC and their correlation with IGF2BP2 expression in TCGA_LIHC and Catholic_mLIHC.**

| No. | Genes | TCGA_LIHC mRNA expression (FPKM) | | | Correlation with *IGF2BP2* expression (Pearson’s *r*) | |
| --- | --- | --- | --- | --- | --- | --- |
|  |  | Non-tumor  (*n* = 50) | Tumor  (*n* = 371) | Fold change  (Tumor/Non-tumor) | TCGA_LIHC | Catholic_mLIHC |
| 1 | *MYBL2* | 0.22 | 4.66 | 21.16 | 0.55 | 0.33 |
| 2 | *CENPF* | 0.09 | 1.68 | 18.92 | 0.48 | 0.32 |
| 3 | *ASPM* | 0.09 | 1.50 | 17.11 | 0.43 | 0.32 |
| 4 | *ANLN* | 0.10 | 1.59 | 16.02 | 0.42 | 0.32 |
| 5 | *BUB1B* | 0.08 | 1.19 | 15.47 | 0.47 | 0.46 |
| 6 | *FOXM1* | 0.18 | 2.65 | 14.82 | 0.43 | 0.30 |
| 7 | *SPC25* | 0.12 | 1.51 | 12.05 | 0.47 | 0.47 |
| 8 | *CCNB1* | 0.54 | 6.39 | 11.80 | 0.49 | 0.31 |
| 9 | *CDCA8* | 0.24 | 2.74 | 11.40 | 0.43 | 0.31 |
| 10 | *RECQL4* | 0.50 | 3.83 | 7.66 | 0.43 | 0.40 |
| 11 | *HELLS* | 0.09 | 0.68 | 7.50 | 0.50 | 0.47 |
| 12 | *ACSL4* | 3.18 | 19.83 | 6.23 | 0.44 | 0.35 |
| 13 | *MCM4* | 1.41 | 5.84 | 4.14 | 0.45 | 0.33 |
| 14 | *HMGA1* | 6.50 | 26.80 | 4.13 | 0.47 | 0.36 |
| 15 | *INCENP* | 0.53 | 1.85 | 3.49 | 0.41 | 0.43 |
| 16 | *SOX12* | 1.35 | 4.64 | 3.44 | 0.51 | 0.37 |
| 17 | *LMNB2* | 1.26 | 4.22 | 3.35 | 0.47 | 0.38 |
| 18 | *STIP1* | 8.18 | 22.73 | 2.78 | 0.56 | 0.43 |
| 19 | *CKAP4* | 10.37 | 27.62 | 2.66 | 0.56 | 0.37 |
| 20 | *PABPC1* | 60.90 | 152.21 | 2.50 | 0.39 | 0.34 |
| 21 | *PODXL2* | 0.65 | 1.61 | 2.48 | 0.30 | 0.42 |
| 22 | *CPD* | 5.11 | 11.94 | 2.34 | 0.40 | 0.32 |
| 23 | *SKP2* | 2.04 | 4.41 | 2.16 | 0.47 | 0.47 |
| 24 | *CBX5* | 1.54 | 3.16 | 2.05 | 0.34 | 0.33 |
| 25 | *KPNB1* | 8.47 | 16.99 | 2.01 | 0.38 | 0.31 |
| 26 | *PRRC2C* | 3.54 | 6.93 | 1.96 | 0.38 | 0.42 |

Abbreviations: *ANLN*, anillin actin binding protein; *ASPM*, abnormal spindle microtubule assembly; *ACSL4*, acyl-CoA synthetase long chain family member 4; *BUB1B*, BUB1 mitotic checkpoint serine/threonine kinase B; Catholic_mLIHC, Catholic University multistage liver hepatocellular carcinoma cohort; *CBX5*, chromobox 5; *CCNB1*, cyclin B1; *CDCA8*, cell division cycle associated 8; *CENPF*, centromere protein F; *CKAP4*, cytoskeleton associated protein 4; *CPD*, carboxypeptidase D; *FOXM1*, forkhead box M1; FPKM, fragments per kilobase of transcript per million mapped reads; *HELLS*, helicase, lymphoid specific; *HMGA1*, high mobility group AT-hook 1; *INCENP*, inner centromere protein; *KPNB1*, karyopherin subunit beta 1; *LMNB2*, lamin B2; *MCM4*, minichromosome maintenance complex component 4; *MYBL2*, MYB proto-oncogene like 2; *PABPC1*, poly(A) binding protein cytoplasmic 1; *PODXL2*, podocalyxin like 2; *PRRC2C*, proline rich coiled-coil 2C; *RECQL4*, RecQ like helicase 4; *SKP2*, S-phase kinase associated protein 2; *SOX12*, SRY-box transcription factor 12; *SPC25*, SPC25 component of NDC80 kinetochore complex; *STIP1*, stress induced phosphoprotein 1; TCGA_LIHC, The Cancer Genome Atlas Liver Hepatocellular Carcinoma.

**Table S3. MYBL2-enriched candidate transcriptional target genes and their differential expression between non-tumor and tumor samples in the TCGA_LIHC.**

| No. | Genes | GSE170799 | TCGA_LIHC mRNA expression (FPKM) | | |
| --- | --- | --- | --- | --- | --- |
|  |  | MYBL2 enrichment  (TPM) | Non-tumor  (*n* = 50) | Tumor  (*n* = 371) | Fold change  (Tumor/Non-tumor) |
| 1 | *HMGB2* | 956.84 | 3.92 | 13.46 | 3.43 |
| 2 | *RACGAP1* | 734.59 | 0.40 | 2.90 | 7.30 |
| 3 | *PLK1* | 716.37 | 0.13 | 1.91 | 14.58 |
| 4 | *BUB1B* | 599.81 | 0.08 | 1.19 | 15.47 |
| 5 | *PRC1* | 516.68 | 0.23 | 2.73 | 12.04 |
| 6 | *SGOL2* | 492.97 | 0.09 | 0.68 | 7.56 |
| 7 | *UBE2C* | 473.25 | 0.40 | 7.57 | 18.78 |
| 8 | *KPNA2* | 437.09 | 4.10 | 15.76 | 3.84 |
| 9 | *NUSAP1* | 425.37 | 1.01 | 6.19 | 6.13 |
| 10 | *CCNB1* | 364.30 | 0.54 | 6.39 | 11.80 |
| 11 | *HMMR* | 337.59 | 0.15 | 1.87 | 12.68 |
| 12 | *CKAP2L* | 323.85 | 0.06 | 0.84 | 12.95 |
| 13 | *CDCA3* | 299.14 | 0.09 | 1.15 | 13.09 |
| 14 | *DBF4* | 297.36 | 0.42 | 1.19 | 2.81 |
| 15 | *CKS2* | 281.31 | 6.59 | 22.86 | 3.47 |
| 16 | *CDC25C* | 277.51 | 0.05 | 1.28 | 25.86 |
| 17 | *DLGAP5* | 257.67 | 0.08 | 1.37 | 16.95 |
| 18 | *NDC80* | 253.33 | 0.14 | 1.82 | 12.93 |
| 19 | *TK1* | 237.00 | 2.21 | 14.07 | 6.37 |
| 20 | *KIF20A* | 231.55 | 0.12 | 2.08 | 16.81 |
| 21 | *TUBA1B* | 221.75 | 8.08 | 21.03 | 2.60 |
| 22 | *TPX2* | 218.28 | 1.14 | 6.11 | 5.38 |
| 23 | *ANLN* | 202.14 | 0.10 | 1.59 | 16.02 |
| 24 | *CCNB2* | 200.23 | 0.17 | 2.83 | 16.53 |
| 25 | *GTSE1* | 166.60 | 0.07 | 1.04 | 15.07 |
| 26 | *KIF2C* | 164.47 | 0.12 | 2.09 | 18.05 |
| 27 | *H2AFX* | 146.51 | 3.83 | 13.73 | 3.58 |
| 28 | *PSRC1* | 137.83 | 0.35 | 1.56 | 4.48 |
| 29 | *AURKB* | 135.34 | 0.20 | 2.72 | 13.36 |
| 30 | *CDCA8* | 126.98 | 0.24 | 2.74 | 11.40 |
| 31 | *SKA3* | 119.70 | 0.08 | 1.20 | 15.68 |
| 32 | *OIP5* | 103.72 | 0.23 | 1.49 | 6.48 |
| 33 | *TOP2A* | 99.16 | 0.27 | 4.74 | 17.51 |
| 34 | *CDC20* | 76.36 | 0.29 | 5.87 | 20.52 |
| 35 | *KIF15* | 75.60 | 0.05 | 0.63 | 13.94 |
| 36 | *LMNB2* | 52.36 | 1.26 | 4.22 | 3.35 |
| 37 | *EZH2* | 39.88 | 0.33 | 2.37 | 7.17 |
| 38 | *CENPH* | 36.12 | 0.35 | 1.77 | 5.12 |
| 39 | *KIF23* | 23.89 | 0.07 | 0.89 | 13.17 |
| 40 | *KIF11* | 16.32 | 0.24 | 1.49 | 6.19 |

Abbreviations: *AURKB*, aurora kinase B; *ANLN*, anillin actin binding protein; *BUB1B*, BUB1 mitotic checkpoint serine/threonine kinase B; *CCNB1*, cyclin B1; *CCNB2*, cyclin B2; *CDC20*, cell division cycle 20; *CDC25C*, cell division cycle 25C; *CDCA3*, cell division cycle associated 3; *CDCA8*, cell division cycle associated 8; *CENPH*, centromere protein H; *CKAP2L*, cytoskeleton associated protein 2 like; *CKS2*, CDC28 protein kinase regulatory subunit 2; *DBF4*, DBF4-CDC7 kinase regulatory subunit; *DLGAP5*, DLG associated protein 5; *EZH2*, enhancer of zeste 2 polycomb repressive complex 2 subunit; FPKM, fragments per kilobase of transcript per million mapped reads; *GTSE1*, G2 and S-phase expressed 1; *H2AFX*, H2A.X variant histone; *HMGB2*, high mobility group box 2; *HMMR*, hyaluronan mediated motility receptor; *KIF11*, kinesin family member 11; *KIF15*, kinesin family member 15; *KIF20A*, kinesin family member 20A; *KIF23*, kinesin family member 23; *KIF2C*, kinesin family member 2C; *KPNA2*, karyopherin subunit alpha 2; *LMNB2*, lamin B2; *NDC80*, NDC80 kinetochore complex component; *NUSAP1*, nucleolar and spindle associated protein 1; *OIP5*, Opa interacting protein 5; *PLK1*, polo like kinase 1; *PRC1*, protein regulator of cytokinesis 1; *PSRC1*, proline and serine rich coiled-coil 1; *RACGAP1*, Rac GTPase activating protein 1; *SGOL2*, shugoshin 2; *SKA3*, spindle and kinetochore associated complex subunit 3; TCGA_LIHC, The Cancer Genome Atlas Liver Hepatocellular Carcinoma; *TK1*, thymidine kinase 1; *TOP2A*, DNA topoisomerase II alpha; *TPX2*, TPX2 microtubule nucleation factor; *TUBA1B*, tubulin alpha 1b; *UBE2C*, ubiquitin conjugating enzyme E2 C.

**Table S4. Classification of liver tumor burden based on the liver weight-to-body weight (LW/BW) ratio.**

| Tumor-burden category | LW/BW ratio (%) |
| --- | --- |
| Normal liver | 4 to 6 |
| Mild tumor burden | 6 to 8 |
| Moderate tumor burden | 8 to 12 |
| Severe tumor burden | 12 to 20 |

Abbreviations: LW/BW, liver weight-to-body weight ratio.

**Table S5. List of siRNAs used in transfection**

| siRNA | Accession No. | Strand | Nucleotide sequence |
| --- | --- | --- | --- |
| Control siRNA | NA | Sense | 5'-CCUACGCCACCAAUUUCGU-3' |
|  |  | Antisense | 5'-ACGAAAUUGGUGGCGUAGG-3' |
| *HMGB2* siRNA | NM_002129.4 | Sense | 5’- UCGCGGAAUUCUCCAAGAAGUGU -3’ |
|  |  | Antisense | 5’- ACACUUCUUGGAGAAUUCCGCGA -3’ |
| *Hmgb2* siRNA | NM_008252.3 | Sense | 5’- GAGCGACAAAGCUCGUUAUGACA -3’ |
|  |  | Antisense | 5’- UGUCAUAACGAGCUUUGUCGCUC -3’ |
| *IGF2BP2* siRNA | NM_001164617.2 | Sense | 5’- GCCCAUUCUCAACAACGCCAAUAUA -3’ |
|  |  | Antisense | 5’- UAUAUUGGCGUUGUUGAGAAUGGGC -3’ |
| *Igf2bp2* siRNA | NM_183029.2 | Sense | 5’- CUGGUAUAGAAAGGAACUU -3’ |
|  |  | Antisense | 5’- AAGUUCCUUUCUAUACCAG -3’ |
| *METTL3* siRNA | NM_019852.5 | Sense | 5’- AGCUACAGAUCCUGAGUUAGAGA -3’ |
|  |  | Antisense | 5’- UCUCUAACUCAGGAUCUGUAGCU -3’ |
| *Mettl3* siRNA | NM_019721.2 | Sense | 5’- CGACUCUUUCCACCUCAGU -3’ |
|  |  | Antisense | 5’- ACUGAGGUGGAAAGAGUCG -3’ |
| *MYBL2* siRNA | NM_002466.4 | Sense | 5’- CACACUGCCCAAGUCUCUA -3’ |
|  |  | Antisense | 5’- UAGAGACUUGGGCAGUGUG -3’ |
| *Mybl2* siRNA | NM_008652.2 | Sense | 5’- GACUCUACUAUCCCACUCU -3’ |
|  |  | Antisense | 5’- AGAGUGGGAUAGUAGAGUC -3’ |

NA, not applicable. Abbreviations: *HMGB2*, high mobility group box 2; *IGF2BP2*, insulin-like growth factor 2 mRNA-binding protein 2; *METTL3*, methyltransferase-like 3; *MYBL2*, MYB proto-oncogene like 2; NA, not applicable; siRNA, small interfering RNA.

**Table S6. List of antibodies used in western blot analysis, immunoprecipitation, and FACS**

| Protein | Manufacturer | Catalog No. | Application | Dilution |
| --- | --- | --- | --- | --- |
| Caspase 3 | Cell signaling | #9668 | Western blot | 1:1000 |
| Cleaved caspase 3 | Cell signaling | #9661 | Western blot | 1:500 |
| CDK1 | Cell signaling | #9112S | Western blot | 1:1000 |
| Cleaved PARP | Cell signaling | #9541 | Western blot | 1:1000 |
| Cyclin A2 | Cell signaling | 4656S | Western blot | 1:1000 |
| Cyclin B1 | Cell signaling | 4138S | Western blot | 1:1000 |
| FLAG | Cell signaling | #2368 | Western blot | 1:1000 |
| GAPDH | Cell signaling | 97166T | Western blot | 1:1000 |
| HMGB2 | Cell signaling | 14163S | Western blot | 1:1000 |
| IGF2BP2 | Cell signaling | #14672 | Western blot | 1:1000 |
| METTL3 | Abcam | 195352 | Western blot | 1:1000 |
| MYBL2 | Santa Cruz | sc-390198 | Western blot | 1:500 |
| PARP | Cell signaling | #9542 | Western blot | 1:1000 |
| RAGE | Cell signaling | 42544S | Western blot | 1:1000 |
| TOP2A | Santa Cruz | sc-56803 | Western blot | 1:500 |
| WEE1 | Santa Cruz | sc-9037 | Western blot | 1:1000 |
| FLAG | Sigma | 080M6035 | Immunoprecipitation | 1:50 |
| IGF2BP2 | Cell signaling | #14672 | Immunoprecipitation | 1:50 |
| m6A | Synaptic Systems | 202 003 | Immunoprecipitation | 1:50 |
| METTL3 | Abcam | 195352 | Immunoprecipitation | 1:50 |
| MYBL2 | Santa Cruz | sc-390198 | Immunoprecipitation | 1:50 |
| CD11b | Bio Legend | 301350 | FACS | NA |
| CD163 | Bio Legend | 333611 | FACS | NA |
| F4/80 | Bio Legend | 123121 | FACS, IF | NA |

Abbreviations: CD11b, cluster of differentiation 11b; CD163, cluster of differentiation 163; CDK1, cyclin-dependent kinase 1; FACS, fluorescence-activated cell sorting; FLAG, FLAG epitope tag; GAPDH, glyceraldehyde-3-phosphate dehydrogenase; HMGB2, high mobility group box 2; IF, immunofluorescence; IGF2BP2, insulin-like growth factor 2 mRNA-binding protein 2; m6A, N6-methyladenosine; METTL3, methyltransferase-like 3; MYBL2, MYB proto-oncogene like 2; NA, not applicable; PARP, poly(ADP-ribose) polymerase; RAGE, receptor for advanced glycation end products; TOP2A, DNA topoisomerase II alpha; WEE1, WEE1 G2 checkpoint kinase.

**Table S7. List of primer sequences used in PCR experiments**

| Gene | Primer | Nucleotide sequence | Experiments |
| --- | --- | --- | --- |
| *CD163* | Forward | 5'- TTGCCAGCAGCTTAAATGTG -3' | qRT-PCR |
|  | Reverse | 5'- TTGCAGTGCCTTCACTTGAG -3' |  |
| *GAPDH* | Forward | 5'- CATCACTGCCACCCAGAAGACTG -3' | qRT-PCR |
|  | Reverse | 5'- ATGCCAGTGAGCTTCCCGTTCAG -3' |  |
| *Gapdh* | Forward | 5'- CCACCCAGAAGACTGTGGAT -3' | qRT-PCR |
|  | Reverse | 5'- CACATTGGGGGTAGGAACAC -3' |  |
| *HMGB2* | Forward | 5'- TTACGTTCCTCCCAAAGGTG -3' | qRT-PCR |
|  | Reverse | 5'- TCTTTGGCTGACTGCTCAGA -3' |  |
| *IL10* | Forward | 5'- TGCCTTCAGCAGAGTGAAGA -3' | qRT-PCR |
|  | Reverse | 5'- GGTCTTGGTTCTCAGCTTGG -3' |  |
| *MSR1* | Forward | 5'- CCTCGTGTTTGCAGTTCTCA -3' | qRT-PCR |
|  | Reverse | 5'- CCATGTTGCTCATGTGTTCC -3' |  |
| *MYBL2* | Forward | 5'- CCAGCCACTTCCCTAACCG -3' | qRT-PCR |
|  | Reverse | 5'- CAGTGTCCACTGCTTTGTGC -3' |  |
| Region A | Forward | 5'- GAGACACTCGGGGCTTGGAATT -3' | ChIP |
|  | Reverse | 5'- GGAGCTGTAGTCGCTCTGCTCT -3' |  |
| Region B | Forward | 5'- AACTCCAGGCTCTCCTCATTTA -3' | ChIP |
|  | Reverse | 5'- GCGGGAAGAGGGTTTGAAAAAT -3' |  |
| Region C | Forward | 5'- TTTACAGCCCCAACCAGTCAGC -3' | ChIP |
|  | Reverse | 5'- ATCTCTAACGCAATCCTCAGCC -3' |  |

Abbreviations: *CD163*, cluster of differentiation 163; ChIP, chromatin immunoprecipitation; *GAPDH*, glyceraldehyde-3-phosphate dehydrogenase; *HMGB2*, high mobility group box 2*; IL10*, interleukin 10; *MSR1*, macrophage scavenger receptor 1; *MYBL2*, MYB proto-oncogene like 2; qRT-PCR, quantitative reverse transcription polymerase chain reaction.

**Table S8. Clinicopathological characteristics of clinical tissue samples used for validation.**

| Patient ID | Sex | Age (years) | Etiology | Background liver disease | Tumor size (cm) | Tumor multiplicity | AJCC TNM stage | Grade | AFP (ng/mL) |
| --- | --- | --- | --- | --- | --- | --- | --- | --- | --- |
| P1 | Male | 52 | HBV | Cirrhotic change | 2.9 × 2.5 | 1 | NA | Edmondson grade I–II | 2.71 |
| P2 | Male | 64 | HBV | Steatosis, chronic interface hepatitis, periportal fibrosis | 12.0 × 7.5 | 1 | T4N | Poorly differentiated adenocarcinoma | 65.70 |
| P3 | Male | 59 | NA | Fatty change and chronic inflammation | 3.9 × 2.7 | 1 | NA | NA | 3.68 |
| P4 | Male | 61 | Cryptogenic | No diagnostic abnormalities in the remaining parenchyma | 10.0 × 8.5 | 1 | NA | Edmondson grade II–III | 2.66 |
| P5 | Male | 69 | Alcohol-related | Cholestasis, diffuse | 8.0 × 7.0 | Multinodular | NA | Edmondson grade II–III | 3.98 |
| P6 | Female | 61 | NA | Metastatic carcinoma | 2.4 × 1.8 | NA | NA | NA | NA |
| P7 | Male | 60 | HBV | Chronic hepatitis | 3.0 × 1.8 | 1 | NA | Edmondson grade III | 5.32 |
| P8 | Male | 73 | Alcohol-related | Cirrhosis | 3.6 × 3.0 | 1 | NA | Edmondson grade II–III | 155.40 |
| P9 | Male | 69 | NA | NA | 11.5 × 8.5 × 6.5 | 1 | NA | NA | 2.37 |
| P10 | Female | 69 | Cryptogenic | Chronic hepatitis | 6.4 × 4.5 | 1 | NA | Edmondson grade II–III | 1.79 |
| P11 | Male | 63 | Cryptogenic | Chronic hepatitis | 7.0 × 6.0 | 1 | T1b | Edmondson grade II | 534.00 |
| P12 | Male | 69 | NA | History of colonic adenocarcinoma | 2.0 × 1.8 | 1 | NA | Moderately differentiated | 1.41 |
| P13 | Male | 66 | NA | Metastatic adenocarcinoma | 2.5 × 1.9;  1.0 × 0.8 | 2 | NA | NA | NA |
| P14 | Female | 67 | Cryptogenic | Mild periportal inflammation | 3.3 × 3.2 | 1 | NA | Moderately differentiated | 1.81 |
| P15 | Male | 63 | NA | NA | 8.0 × 6.2 × 6.0 | 1 | NA | NA | 3.52 |
| P16 | Male | 60 | Alcohol-related | Mild cirrhotic change | 3.8 × 3.0 × 2.3 | 1 | NA | Moderately differentiated | 820.80 |
| P17 | Female | 68 | Alcohol-related | Steatohepatitis; chronic hepatitis | 3.8 × 3.3 | 1 | pT1b | Edmondson grade II–III | 2.56 |
| P18 | Female | 55 | HBV | NA | 2.6 × 2.4 | 1 | NA | Moderately differentiated | 50.28 |
| P19 | Male | 68 | HBV | Chronic hepatitis, HBV-associated | 3.0 × 2.5 | 1 | NA | Edmondson grade II | 2.05 |
| P20 | Female | 43 | HBV | Mild portal inflammation | 2.9 × 2.1 | 1 | NA | Edmondson grade I–II | 48.26 |

Abbreviations: AFP, alpha-fetoprotein; AJCC, American Joint Committee on Cancer; HBV, hepatitis B virus; NA, not applicable; TNM, tumor–node–metastasis.

**Supplementary Figure & Figure legends**

**Figure S1. *IGF2BP2* and *MYBL2* are upregulated and associated with poor prognosis in HCC.**

(**A**) Expression of IGF2BP family members in non-tumor (N) and tumor (T) samples from the TCGA_LIHC (N, *n* = 50; T, *n* = 371), ICGC_LIRI (N, *n* = 202; T, *n* = 243), and GSE77314 (N, *n* = 50; T, *n* = 50) cohorts. (**B**) Expression of *IGF2BP1* and *IGF2BP3* across the multistage progression of HCC in the Catholic_mLIHC cohort (non-tumor, *n* = 15; chronic hepatitis, *n* = 20; cirrhosis, *n* = 10; dysplastic nodule, *n* = 17; early HCC, *n* = 11; advanced HCC, *n* = 45). (**C**) Venn diagram showing genes commonly enriched in IGF2BP1, IGF2BP2, and IGF2BP3 RIP-seq datasets (GSE90639). (**D**) Kaplan–Meier analysis of overall survival according to *IGF2BP1* (low, *n* = 128; high, *n* = 127), *IGF2BP2* (low, *n* = 128; high, *n* = 128), and *IGF2BP3* (low, *n* = 128; high, *n* = 123) mRNA expression in TCGA_LIHC using GEPIA2. Patients were stratified into high and low expression groups using the median expression level of each gene as the cutoff. (**E**) Stepwise filtering strategy to identify IGF2BP2-dependent m6A-modified genes in HCC. Genes differentially expressed following *IGF2BP2* knockdown in GSE90684 were further filtered by IGF2BP2 RIP-seq enrichment in GSE90639, differential expression between tumor and non-tumor liver tissues in TCGA_LIHC and Catholic_mLIHC, m6A modification prediction using RMVar, and positive correlation with *IGF2BP2* expression in TCGA_LIHC and Catholic_mLIHC. (**F–G**) Differential expression of *MYBL2* in the ICGC_LIRI and GSE77314 validated by unpaired and paired analyses. (**H**) Differential expression of *MYBL2* in the Catholic_mLIHC cohort. (**I–J**) *MYBL2* mRNA expression across the multistage progression of HCC in TCGA_LIHC (non-tumor, *n* = 50; early HCC, *n* = 51; advanced HCC, *n* = 303) and Catholic_mLIHC cohorts (non-tumor, *n* = 15; chronic hepatitis, *n* = 20; cirrhosis, *n* = 10; dysplastic nodule, *n* = 17; early HCC, *n* = 11; advanced HCC, *n* = 45). (**K**) Pan-cancer analysis of *MYBL2* mRNA expression between non-tumor and tumor samples across TCGA cohorts. All data are shown as the mean ± SEM, **P* < 0.05, ***P* < 0.01, ****P* < 0.001; paired or unpaired t test. Abbreviations: BLCA, bladder urothelial carcinoma; BRCA, breast invasive carcinoma; Catholic_mLIHC, Catholic University multistage liver hepatocellular carcinoma cohort; CESC, cervical squamous cell carcinoma and endocervical adenocarcinoma; CHOL, cholangiocarcinoma; COAD, colon adenocarcinoma; ESCA, esophageal carcinoma; FPKM, fragments per kilobase of transcript per million mapped reads; GBM, glioblastoma multiforme; HCC, hepatocellular carcinoma; HNSC, head and neck squamous cell carcinoma; ICGC, International Cancer Genome Consortium; *IGF2BP1*, insulin-like growth factor 2 mRNA-binding protein 1; *IGF2BP2*, insulin-like growth factor 2 mRNA-binding protein 2; *IGF2BP3*, insulin-like growth factor 2 mRNA-binding protein 3; KICH, kidney chromophobe; KIRC, kidney renal clear cell carcinoma; KIRP, kidney renal papillary cell carcinoma; LIHC, liver hepatocellular carcinoma; LIRI, liver cancer–RIKEN, Japan; LUAD, lung adenocarcinoma; LUSC, lung squamous cell carcinoma; m6A, N6-methyladenosine; *MYBL2*, MYB proto-oncogene like 2; N, non-tumor; PRAD, prostate adenocarcinoma; READ, rectum adenocarcinoma; RIP-seq, RNA immunoprecipitation sequencing; RMVar, RNA modification-associated variant database; SARC, sarcoma; STAD, stomach adenocarcinoma; T, tumor; TCGA, The Cancer Genome Atlas; UCEC, uterine corpus endometrial carcinoma.

**Figure S2. m6A-dependent IGF2BP2 stabilizes *MYBL2* in HCC.**

(**A**) Pearson correlation analysis of *IGF2BP2 and MYBL2* mRNA expression in HCC datasets (TCGA_LIHC, Catholic_mLIHC, ICGC_LIRI and GSE77314). (**B**) qRT-PCR analysis of *MYBL2* mRNA expression in HCC cell lines. Expression levels were compared with those in MIHA cells. (**C**) Western blot analysis of METTL3, IGF2BP2, and MYBL2 protein levels in the hepatic and HCC cell lines, with GAPDH used as a loading control. (**D**) Time-dependent changes in *MYBL2* mRNA expression following *IGF2BP2* knockdown in HCC cells, as measured by qRT-PCR. (**E**) Actinomycin D chase analysis of *MYBL2* mRNA stability in SNU-182 and Hep3B cells transfected with siCtrl or si*IGF2BP2*. The remaining *MYBL2* mRNA was measured by qRT-PCR at 0, 2, 4, 6, and 8 h after actinomycin D treatment. (**F**) RNA immunoprecipitation (RIP)–qPCR showing enrichment of *MYBL2* mRNA in METTL3, IGF2BP2, and m6A immunoprecipitates. (**G**) IGF2BP2 RIP–qPCR analysis of *MYBL2* mRNA in HCC cells transfected with control siRNA or *siMETTL3*, with IgG used as a negative control. (**H**) Western blot analysis of MYBL2 and HMGB2 expression in Hep3B cells following *METTL3*, *IGF2BP2*, or *MYBL2* knockdown. Densitometric quantification of MYBL2 and HMGB2 protein levels normalized to GAPDH is shown on the right. All data are shown as the mean ± SEM, **P* < 0.05, ***P* < 0.01, ****P* < 0.001; paired or unpaired t test. Abbreviations: FPKM, fragments per kilobase of transcript per million mapped reads; GAPDH, glyceraldehyde-3-phosphate dehydrogenase; HCC, hepatocellular carcinoma; *HMGB2*, high mobility group box 2; ICGC, International Cancer Genome Consortium; *IGF2BP2*, insulin-like growth factor 2 mRNA-binding protein 2; IgG, immunoglobulin G; LIHC, liver hepatocellular carcinoma; m6A, N6-methyladenosine; *METTL3*, methyltransferase-like 3; *MYBL2*, MYB proto-oncogene like 2; qRT-PCR, quantitative reverse transcription polymerase chain reaction; RIP, RNA immunoprecipitation; SEM, standard error of the mean; siCtrl, control small interfering RNA; siRNA, small interfering RNA; TCGA, The Cancer Genome Atlas.

**Figure S3. MYBL2 contributes to hepatocarcinogenesis.**

(**A**) Cell growth was assessed by MTT assay in HCC cells transfected with siCtrl or si*MYBL2*. (**B**) Cell proliferation of SNU-182 and Hep3B cells transfected with siCtrl or si*MYBL2* was evaluated by BrdU assay. (**C**) Cell cycle distribution was analyzed by flow cytometry following *MYBL2* knockdown. Representative DNA content histograms (left) and quantification of cells in G1, S, and G2/M phases (right) are shown. Asterisks indicate a significant difference in the G2/M phase population between siCtrl and si*MYBL2*. (**D**) Apoptosis after *MYBL2* knockdown was assessed by Annexin V-FITC/PI staining followed by flow cytometry. Representative flow-cytometry plots and quantification of Annexin V-positive cells are shown. (**E**) Western blot analysis of cell cycle regulatory proteins in HCC cells transfected with siCtrl or si*MYBL2*. GAPDH was used as a loading control. (**F**) Western blot analysis of apoptosis-associated markers in HCC cells. (**G**) Clonogenic assay of HCC cells following *MYBL2* knockdown. Representative colony images and quantification of colony numbers are shown. All data are shown as the mean ± SEM, **P* < 0.05, ***P* < 0.01, ****P* < 0.001; unpaired t test. Abbreviations: BrdU, 5-bromo-2′-deoxyuridine; CDK1, cyclin-dependent kinase 1; FITC, fluorescein isothiocyanate; G1, gap 1 phase; G2/M, gap 2/mitosis phase; GAPDH, glyceraldehyde-3-phosphate dehydrogenase; HCC, hepatocellular carcinoma; MTT, 3-(4,5-dimethylthiazol-2-yl)-2,5-diphenyltetrazolium bromide; *MYBL2*, MYB proto-oncogene like 2; PARP, poly(ADP-ribose) polymerase; PI, propidium iodide; S, synthesis phase; SEM, standard error of the mean; siCtrl, control small interfering RNA; si*MYBL2*, *MYBL2*-targeting small interfering RNA; TOP2A, DNA topoisomerase II alpha; WEE1, WEE1 G2 checkpoint kinase.

**Figure S4. MYBL2 restoration rescues the anti-tumor effects of IGF2BP2 depletion in HCC.**

(**A**) Cell growth was assessed by MTT assay in Hep3B cells transfected with siCtrl or si*IGF2BP2*, and the effects of *IGF2BP2* knockdown were rescued by MYBL2 (MYBL2–OE). (**B**) Cell proliferation was evaluated by BrdU assay in Hep3B cells following *IGF2BP2* knockdown with or without MYBL2 overexpression. (**C**) Clonogenic assay following *IGF2BP2* knockdown with MYBL2 rescue. Representative colony images and quantification of colony numbers are shown. (**D**) Cell cycle distribution was analyzed by PI–based flow cytometry in HCC cells. Representative DNA content histograms are shown (left), and the proportions of cells in G1, S, and G2/M phases are quantified (right). Asterisks indicate a significant difference in the G2/M phase population between si*IGF2BP2* + Vector and si*IGF2BP2* + MYBL2–OE. (**E**) Western blot analysis of cell cycle regulatory proteins in Hep3B cells. FLAG denotes the FLAG (DYK) epitope tag fused to the MYBL2 overexpression construct and was used to verify ectopic MYBL2 expression. GAPDH was used as a loading control. **(F**) Apoptosis of SNU-182 and Hep3B cells was assessed by Annexin V-FITC/PI staining followed by flow cytometry. (**G**) Western blot analysis of apoptosis-associated markers in Hep3B cells. Quantitative data are presented as the mean ± SEM of three independent experiments. Statistical significance was determined using one-way or two-way ANOVA, as appropriate. **P* < 0.05, ***P* < 0.01, and ****P* < 0.001. Abbreviations: ANOVA, analysis of variance; BrdU, 5-bromo-2′-deoxyuridine; CDK1, cyclin-dependent kinase 1; FITC, fluorescein isothiocyanate; G1, gap 1 phase; G2/M, gap 2/mitosis phase; GAPDH, glyceraldehyde-3-phosphate dehydrogenase; HCC, hepatocellular carcinoma; *IGF2BP2*, insulin-like growth factor 2 mRNA-binding protein 2; MTT, 3-(4,5-dimethylthiazol-2-yl)-2,5-diphenyltetrazolium bromide; *MYBL2*, MYB proto-oncogene like 2; OE, overexpression; PARP, poly(ADP-ribose) polymerase; PI, propidium iodide; S, synthesis phase; SEM, standard error of the mean; siCtrl, control small interfering RNA; si*IGF2BP2*, *IGF2BP2*-targeting small interfering RNA; siRNA, small interfering RNA; TOP2A, DNA topoisomerase II alpha; WEE1, WEE1 G2 checkpoint kinase.

**Figure S5. Clinical relevance and tumorigenic potential of HMGB2 in HCC.**

(**A**) Stepwise filtering strategy to identify *HMGB2* as a downstream target of MYBL2 in HCC. MYBL2-enriched genes identified by ChIP-seq (GSE170799) were filtered for differential expression between HCC and non-tumor liver tissues in the TCGA_LIHC and Catholic_mLIHC cohorts, differential expression following *MYBL2* knockdown in GSE90684, and significant correlation with *MYBL2* expression in TCGA_LIHC and Catholic_mLIHC. Candidate transcription-factor target genes were subsequently identified using the Cistrome Cancer and ChEA databases. (**B**) *HMGB2* mRNA expression in non-tumor (*n* = 15) and tumor (*n* = 56) samples from the Catholic_mLIHC cohort. (**C**) *HMGB2* mRNA expression across HCC stages in the TCGA_LIHC cohort (non-tumor, *n* = 50; early HCC, *n* = 51; advanced HCC, *n* = 303), GSE101685 dataset (non-tumor, *n* = 8; early HCC, *n* = 8; advanced HCC, *n* = 16), and Catholic_mLIHC (non-tumor, *n* = 15; chronic hepatitis, *n* = 20; cirrhosis, *n* = 10; dysplastic nodule, *n* = 17; early HCC, *n* = 11; advanced HCC, *n* = 45) cohort. (**D**) Pan-cancer analysis of *HMGB2* mRNA expression between non-tumor and tumor samples across TCGA cohorts. (**E**) Kaplan–Meier analysis of overall survival according to *HMGB2* expression in TCGA_LIHC. Patients were classified into high and low expression groups using the mean *HMGB2* mRNA expression value of the cohort as the cutoff. Survival distributions were compared using the log-rank test, and the number at risk is shown below the survival curve. (**F**) Cell growth was assessed by MTT assay in HCC cells transfected with siCtrl or si*HMGB2*. Absorbance was measured at 570 nm. (**G**) Cell proliferation of SNU-182 and Hep3B cells was evaluated by BrdU assay. (**H**) Clonogenic assay following *HMGB2* knockdown. Representative colony images and quantification of colony numbers are shown. Quantitative data are presented as the mean ± SEM of three independent experiments. Statistical significance was determined using one-way or two-way ANOVA, as appropriate. **P* < 0.05, ***P* < 0.01, and ****P* < 0.001. Abbreviations: ANOVA, analysis of variance; BLCA, bladder urothelial carcinoma; BRCA, breast invasive carcinoma; BrdU, 5-bromo-2′-deoxyuridine; CESC, cervical squamous cell carcinoma and endocervical adenocarcinoma; ChIP-seq, chromatin immunoprecipitation sequencing; CHOL, cholangiocarcinoma; Cistrome, chromatin immunoprecipitation sequencing and chromatin accessibility database; COAD, colon adenocarcinoma; ESCA, esophageal carcinoma; FDR, false discovery rate; FPKM, fragments per kilobase of transcript per million mapped reads; GBM, glioblastoma multiforme; HCC, hepatocellular carcinoma; *HMGB2*, high mobility group box 2; HNSC, head and neck squamous cell carcinoma; KIRC, kidney renal clear cell carcinoma; LIHC, liver hepatocellular carcinoma; LUAD, lung adenocarcinoma; LUSC, lung squamous cell carcinoma; MTT, 3-(4,5-dimethylthiazol-2-yl)-2,5-diphenyltetrazolium bromide; *MYBL2*, MYB proto-oncogene like 2; NT, non-tumor; SEM, standard error of the mean; siCtrl, control small interfering RNA; si*HMGB2*, *HMGB2*-targeting small interfering RNA; siRNA, small interfering RNA; STAD, stomach adenocarcinoma; TCGA, The Cancer Genome Atlas; UCEC, uterine corpus endometrial carcinoma.

**Figure S6. MYBL2 directly regulates *HMGB2* transcription in HCC cells.**

(**A**) Pearson correlation analysis between *MYBL2* and *HMGB2* mRNA expression in the TCGA_LIHC, Catholic_mLIHC, ICGC_LIRI, and GSE77314. (**B**) *HMGB2* mRNA expression in Hep3B cells transfected with siCtrl, si*IGF2BP2*, or si*MYBL2*, as measured by qRT-PCR. (**C**) Western blot analysis of intracellular and secreted HMGB2 protein levels in Hep3B cells following *MYBL2* knockdown. GAPDH was used as a loading control, and Coomassie blue staining is shown for conditioned-medium proteins. (**D**) Chromatin immunoprecipitation (ChIP) was performed using an anti-MYBL2 antibody in Hep3B cells, followed by qPCR analysis of the indicated HMGB2 promoter regions. (**E**) Dual-luciferase reporter assay in Hep3B cells co-transfected with *HMGB2* promoter constructs and either si*MYBL2* or pcDNA3.1_MYBL2. Luciferase activity was normalized to Renilla activity. (**F**) Relative mRNA expression of *IGF2BP2*, *MYBL2*, and *HMGB2* in 20 pairs of primary human HCC tissues and matched adjacent non-cancerous liver tissues, as determined by qRT-PCR. (**G**) Western blot analysis of IGF2BP2, MYBL2, and HMGB2 protein expression in the same 20 pairs of primary human HCC tissues and matched adjacent non-cancerous liver tissues. GAPDH was used as a loading control. Red asterisks indicate non-concordant pairs in which not all three proteins were increased in tumor tissues compared with matched non-cancerous tissues. Quantitative data are presented as the mean ± SEM of three independent experiments. Statistical significance was determined using one-way or two-way ANOVA, as appropriate. **P* < 0.05, ***P* < 0.01, and ****P* < 0.001. Abbreviations: ANOVA, analysis of variance; ChIP, chromatin immunoprecipitation; GAPDH, glyceraldehyde-3-phosphate dehydrogenase; HCC, hepatocellular carcinoma; *HMGB2*, high mobility group box 2; ICGC, International Cancer Genome Consortium; IGF2BP2, insulin-like growth factor 2 mRNA-binding protein 2; IgG, immunoglobulin G; LIHC, liver hepatocellular carcinoma; *MYBL2*, MYB proto-oncogene like 2; qRT-PCR, quantitative reverse transcription polymerase chain reaction; SEM, standard error of the mean; siCtrl, control small interfering RNA; si*IGF2BP2*, *IGF2BP2*-targeting small interfering RNA; si*MYBL2*, *MYBL2*-targeting small interfering RNA; TCGA, The Cancer Genome Atlas.

**Figure S7. HMGB2–RAGE signaling suppresses macrophage-mediated tumor-cell phagocytosis in HCC.**

(**A**) Relative immune-cell composition in the TCGA_LIHC cohort by CIBERSORTx. (**B**) Phagocytosis of Hep3B cells by M1-polarized THP-1–derived macrophages was assessed following *HMGB2* knockdown. Representative fluorescence images (top) and flow cytometric quantification of phagocytic uptake (bottom) are shown. The bar graph indicates the percentage of phagocytosis-positive macrophages. (**C**) Apoptosis was assessed by Annexin V-FITC/propidium iodide (PI) staining followed by flow cytometry in SNU-182 and Hep3B cells transfected with si*HMGB2*. (**D**) Co-immunoprecipitation analysis was performed to assess the interaction between tumor-derived HMGB2 and macrophage RAGE. HCC cells were transfected with FLAG-tagged HMGB2 (pcDNA3.1_HMGB2), and CM was applied to THP-1–derived macrophages prior to immunoprecipitation. (**E**) Phagocytosis was measured by flow cytometry after si*MYBL2* transfection, with modulation by recombinant HMGB2 (rHMGB2; 500 ng) and RAGE antagonist peptide (RAP; 10 μmol/L) during macrophage–tumor cell co-culture. (**F**) Flow cytometry-based analysis of THP-1-derived macrophage-mediated phagocytosis of Hep3B and SNU-182 cells following *IGF2BP2* depletion, with or without recombinant HMGB2 treatment. Representative flow cytometry plots and quantification of phagocytosis are shown. (**G**) Mouse HCC cells transfected with siCtrl, si*Mybl2*, or si*Hmgb2* were co-cultured with mouse primary peritoneal macrophages, and phagocytosis was quantified by flow cytometry. (**H**) Phagocytosis of human HCC cells by mouse primary peritoneal macrophages. Representative flow cytometry plots and quantification of the F4/80-positive, CFSE-positive macrophage population are shown. (**I**) The gating of phagocytosis in macrophages alone. (**J**) The gating of phagocytosis in macrophages co-cultured with CFSE-labeled cancer cells. All data are shown as the mean ± SEM, **P* < 0.05, ***P* < 0.01, ****P* < 0.001; unpaired t test. Abbreviations: APC, allophycocyanin; CD11b, cluster of differentiation 11b; CFSE, carboxyfluorescein succinimidyl ester; CIBERSORTx, Cell-type Identification By Estimating Relative Subsets Of RNA Transcripts; CM, conditioned medium; Co-IP, co-immunoprecipitation; FITC, fluorescein isothiocyanate; HCC, hepatocellular carcinoma; *HMGB2*, high mobility group box 2; *IGF2BP2*, insulin-like growth factor 2 mRNA-binding protein 2; LIHC, liver hepatocellular carcinoma; MYBL2, MYB proto-oncogene like 2; PI, propidium iodide; RAGE, receptor for advanced glycation end products; RAP, RAGE antagonist peptide; rHMGB2, recombinant HMGB2; SEM, standard error of the mean; siCtrl, control small interfering RNA; si*HMGB2*, *HMGB2*-targeting small interfering RNA; si*IGF2BP2*, *IGF2BP2*-targeting small interfering RNA; si*MYBL2*, *MYBL2*-targeting small interfering RNA; TCGA, The Cancer Genome Atlas.

**Figure S8. *In vivo* validation of the IGF2BP2**–**MYBL2**–**HMGB2 axis in hepatocarcinogenesis.**

(**A**) Timeline of *in vivo* siRNA administration in H-*ras*12V homozygous transgenic (*Ras*-Tg) mice. Mice were assigned to the indicated treatment groups and administered siRNA by weekly tail-vein injection, with serial ultrasonography performed at the indicated time points to monitor liver tumor development (*n* = 5 per group). (**B**) The number of liver tumor masses in each mouse treated with siCtrl, si*Mettl3*, si*Igf2bp2*, or si*Mybl2* at the indicated weeks of age is shown in the table. (**C**) Liver weight-to-body weight ratio in each group. Background shading indicates the predefined LW/BW ratio-based categories of liver tumor burden: normal liver, 4%–6%; mild tumor burden, 6%–8%; and moderate tumor burden, 8%–12%. (**D**) Western blot analysis of Mettl3, Igf2bp2, Mybl2 expression in liver tissues from H-*ras* transgenic mice treated with siCtrl, si*Mettl3*, or si*Igf2bp2*. Gapdh served as a loading control. (**E**) Western blot analysis of Mybl2 and Hmgb2 protein expression in liver tissues from H-*ras* transgenic mice treated with siCtrl or siMybl2. Gapdh was used as a loading control. (**F**) Representative F4/80 immunofluorescence images and quantification of F4/80-positive macrophages in liver tumor tissues from H-*ras* transgenic mice treated with siCtrl, siHmgb2, or the RAGE antagonist peptide (RAP). F4/80 is shown in red, and nuclei were counterstained with DAPI in blue. F4/80-positive macrophages were quantified as the number of positive cells per square millimeter of tumor tissue. Quantitative data are presented as the mean ± SEM. Statistical significance was determined using one-way or two-way ANOVA, as appropriate. **P* < 0.05, ***P* < 0.01, and ****P* < 0.001. Abbreviations: ANOVA, analysis of variance; DAPI, 4′,6-diamidino-2-phenylindole; GAPDH, glyceraldehyde-3-phosphate dehydrogenase; HCC, hepatocellular carcinoma; *HMGB2*, high mobility group box 2; *IGF2BP2*, insulin-like growth factor 2 mRNA-binding protein 2; LW/BW, liver weight-to-body weight ratio; *METTL3*, methyltransferase-like 3; *MYBL2*, MYB proto-oncogene like 2; RAGE, receptor for advanced glycation end products; RAP, RAGE antagonist peptide; *Ras*-Tg, H-*ras*12V homozygous transgenic mice; SEM, standard error of the mean; siCtrl, control small interfering RNA; si*Hmgb2*, *Hmgb2*-targeting small interfering RNA; si*Igf2bp2*, *Igf2bp2*-targeting small interfering RNA; si*Mettl3*, *Mettl3*-targeting small interfering RNA; si*Mybl2*, *Mybl2*-targeting small interfering RNA; siRNA, small interfering RNA.
